# LETHE ‐ A digitally supported multimodal lifestyle program to promote brain health among older adults: Study design, progress, and first results

**DOI:** 10.1002/alz.088661

**Published:** 2025-01-09

**Authors:** Anna Rosenberg, Helena Untersteiner, Anna Giulia Guazzarini, Markus Bödenler, Jeroen Bruinsma, Bianca Schnalzer, Matteo Colombo, Rik Crutzen, Ana Diaz, Dimitrios Fotiadis, Hannes Hilberger, Simone Huber, Nico Kaartinen, Miia Kivipelto, Jenni Lehtisalo, Vassilis Loukas, Jyrki Lötjönen, Mattia Pirani, Charlotta Thunborg, Sten Hanke, Francesca Mangialasche, Patrizia Mecocci, Elisabeth Stögmann, Tiia Ngandu, LETHE consortium

**Affiliations:** ^1^ Finnish Institute for Health and Welfare, Helsinki Finland; ^2^ Karolinska Institutet, Stockholm Sweden; ^3^ Medical University of Vienna, Vienna Austria; ^4^ University of Perugia, Perugia Italy; ^5^ FH JOANNEUM University of Applied Sciences, Graz Austria; ^6^ Maastricht University, Maastricht Netherlands; ^7^ i2Grow, Bologna Italy; ^8^ Alzheimer Europe, Luxembourg Luxembourg; ^9^ University of Ioannina, Ioannina Greece; ^10^ Biomedical Research Institute, FORTH, Ioannina Greece; ^11^ FH Joanneum University of Applied Sciences, Graz Austria; ^12^ Kaasa Solution GmbH, Dusseldorf Germany; ^13^ Institute of Public Health, University of Eastern Finland, Kuopio Finland; ^14^ Theme Inflammation and Aging, Karolinska University Hospital, Stockholm Sweden; ^15^ Imperial College London, London United Kingdom; ^16^ University of Eastern Finland, Kuopio Finland; ^17^ Population Health Unit Finnish Institute for Health and Welfare, Helsinki Finland; ^18^ Combinostics Ltd, Tampere Finland; ^19^ Population Health Unit, Finnish Institute for Health and Welfare, Helsinki Finland

## Abstract

**Background:**

The Finnish Geriatric Intervention Study to Prevent Cognitive Impairment and Disability (FINGER) multimodal lifestyle intervention yielded cognitive and other health benefits in older adults at risk of cognitive decline. The two‐year multinational randomized controlled LETHE trial evaluates the feasibility of a digitally supported, adapted FINGER intervention among at‐risk older adults. Technology is used to complement in‐person activities, for the intervention delivery, personalize recommendations, and collect digital biomarkers.

**Method:**

Trial includes older adults (60‐77 years) with digital readiness and increased dementia risk but without substantial cognitive impairment. Participants are enrolled at four sites (Austria, Finland, Italy, Sweden). At baseline, participants were randomized 1:1 ratio to 1) intervention i.e., structured multimodal lifestyle program (including diet, exercise, cognitive training, vascular/metabolic risk management, social stimulation, sleep/stress management) where in‐person activities led by professionals are supported with an Android mobile phone application developed by the consortium (the LETHE App); or 2) control i.e., self‐guided program (regular health advice, simplified App with no personalized/interactive content). All participants wear smartwatches to gather passive data (e.g., physical activity, sleep). Primary outcomes are retention, adherence, and change in validated dementia risk scores. Secondary outcomes include changes in lifestyle, cognition, stress, sleep, health‐related quality of life, health literacy. Additional outcomes (exploratory) include e.g., participant experiences and dementia‐related biomarkers (Alzheimer’s disease blood markers, neuroimaging). A sub‐study explores the feasibility of novel interactive technology (audio glasses, social robot).

**Results:**

Recruitment began in September 2022, and the last participant was randomized in June 2023. In total, 156 individuals were randomized (mean age 69 years, 65% women; balanced recruitment across the four sites). Vascular and lifestyle risk factors were common (e.g., 65% with hypertension, 69% with hypercholesterolemia, 39% physically inactive), indicating successful recruitment of a population with risk reduction potential. Trial will be completed by summer 2025. Retention until the first post‐baseline visit at 6 months is high (n = 2 discontinued, retention 98.7%). Latest progress of the trial will be presented.

**Conclusion:**

LETHE provides crucial information about the feasibility of technology and a digitally supported FINGER program to promote brain health. Digital tools specifically designed for older adults could offer potential for large‐scale, cost‐effective prevention programs.

